# Response to nab-paclitaxel and nedaplatin in a heavily-metastatic thymic carcinoma: A case report

**DOI:** 10.3892/ol.2015.2953

**Published:** 2015-02-10

**Authors:** PING ZHAN, HAIYAN XIE, LI-KE YU

**Affiliations:** 1 Department of Respiratory Medicine, Nanjing Chest Hospital, Nanjing, Jiangsu, P.R. China; 2 Nanjing Clinical Center of Respiratory Diseases, Nanjing, Jiangsu, P.R. China

**Keywords:** thymic carcinoma, chemotherapy, molecular target therapy, nab-paclitaxel

## Abstract

Metastatic thymic carcinoma is an aggressive cancer that usually responds poorly to multimodal therapies. Although surgical resection is the preferred treatment for patients with advanced or metastatic disease, the clinical prognosis is typically poor. The present study describes a 63-year-old patient with thymic carcinoma who underwent a range of antitumor treatments, including surgical resection, post-operative radiotherapy and post-operative chemotherapy with several drugs, but ultimately responded to treatment with nab-paclitaxel (nab-P) and nedaplatin. Subsequent to six cycles of nab-P and nedaplatin, the lung and peritoneal metastases decreased in size and the pleural effusion was reduced. To the best of our knowledge, this is the first study to describe the response of an advanced thymic carcinoma to nab-P chemotherapy.

## Introduction

Thymic epithelial tumors are a rare form of neoplasm consisting of two primary histological types: Thymoma and thymic carcinomas (1). The lesions form a rare category of heterogeneous lesions, presenting with a large range of pathological and clinical characteristics. Thymomas are the most frequently diagnosed primary anterior mediastinal tumors, with an incidence of 1.5 cases per million individuals (2). Although thymic carcinomas are rarer than thymomas, they are more invasive and have a poorer prognosis. The five-year survival rates for all stages of thymoma and thymic carcinomas are 78 and 40%, respectively (3–5).

Surgical resection, followed by radiation therapy, are the preferred treatments for all subtypes of thymic carcinoma without distant metastasis. Patients with unresectable tumors or metastasis, however, are treated with systemic chemotherapy. Due to the rarity of the disease, a standard chemotherapy regimen for thymic carcinoma is yet to be established (6).

The present study describes a 63-year-old female with thymic carcinoma who underwent a range of antitumor treatments, including surgical resection, post-operative radiotherapy, post-operative chemotherapy with gemcitabine-carboplatin (GC) and gemcitabine-cisplatin (GP), second-line chemotherapy with docetaxel and cisplatin (DP) following relapse, tyrosine kinase inhibitor (TKI) treatment, and finally chemotherapy with nab-paclitaxel (nab-P) and nedaplatin. The patient demonstrated a good response to nab-P and remains alive three years post-treatment. Written informed consent was obtained from the patient.

## Case report

**Figure 1:**
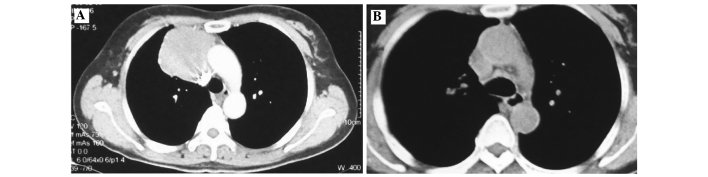
Thoracic computed tomography images (A) prior to and (B) subsequent to surgery.

**Figure 2:**
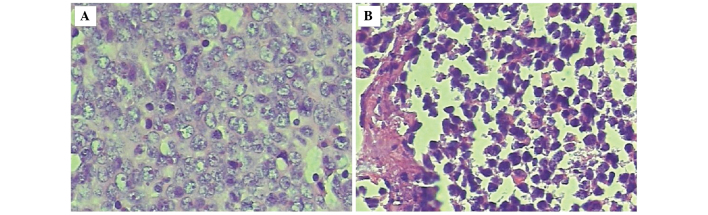
Pathological features of (A) the thymic carcinoma tissue and (B) the metastatic tissue identified in the lung (stain, hematoxylin and eosin; magnification, ×400).

In August 2011, a 63-year-old female underwent thoracic computed tomography (CT) and was diagnosed with a mass in the upper right mediastinum that measured 7.5×7 cm and was in close proximity to the superior vena cava (Fig. 1A). The patient did not present with any respiratory symptoms, such as a cough or chest pain. Complete resection of the tumor was performed under general anesthetic in September 2011 (Fig. 1B). During surgery, tumor cell invasion into the superior vena cava and left innominate vein, and pericardial adhesion were observed. The pathological evidence established a diagnosis of thymic squamous cell carcinoma (Fig. 2A), with positive lymph nodes under the clavicle. Treatment with GP (1.4 mg gemcitabine, days 1 and 8; 100 mg cisplatin, day 1; one cycle lasting 21 days) and GC (1.4 mg gemcitabine, days 1 and 8; 400 mg carboplatin, day 1; three cycles lasting 63 days). Subsequent to chemotherapy, the patient received post-operative radiotherapy to the mediastinum with a total dosage of 56 Gy (2 Gy/day over 28 days).

**Figure 3:**
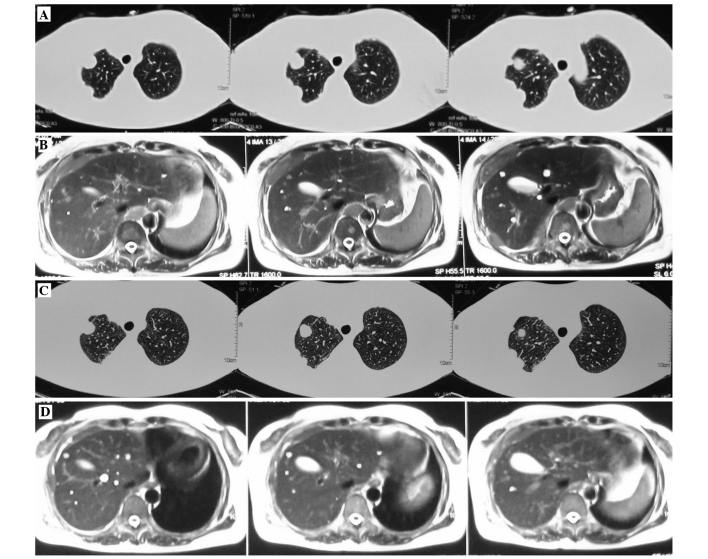
(A) Thoracic computed tomography (CT) images prior to nab-paclitaxel chemotherapy. (B) Abdominal magnetic resonance imaging (MRI) revealing the presence of peritoneal metastasis prior to nab-paclitaxel chemotherapy. (C) Thoracic CT revealing that the metastatic lesion in the lung was reduced in size following nab-paclitaxel chemotherapy. (D) Abdominal MRI revealing that the metastatic lesion in the peritoneum was significantly reduced following nab-paclitaxel chemotherapy.

At the follow-up visit, the patient had no discomfort and the disease had not progressed. However, in late February 2013, thoracic CT revealed several unequal nodules in the right lung and under the pleura. The disease had recurred following 18 months of progression-free survival. Subsequent to two cycles of chemotherapy with DP (docetaxel, 100 mg on day 1; cisplatin, 100 mg on day 1 for two cycles lasting 42 days), the patient was reexamined. This time, thoracic CT revealed the presence of metastatic lesions in the right lung, which were larger than those previously observed and were considered unsuitable for DP therapy. Paraffin-embedded sections of the thymic carcinoma were then analyzed for the 18–21 mutation of the EGFR gene. The results of the amplification refractory mutation system (ARMS) revealed a mutation in L858R of exon 21. The patient was administered gefitinib and a month later was reexamined by thoracic CT. The results indicated progression of the lesion and so treatment was stopped. During the three-month, drug-free observation period, the metastatic lesion in the right lung significantly progressed (Fig. 3A). Furthermore, right-sided pleural effusion, evidence of peritoneal metastasis (Fig. 3B), and symptoms of chest distress and shortness of breath were evident. On August 16, 2013, a percutaneous intrathoracic lung biopsy was performed following a CT scan. The pathological evidence indicated the presence of squamous cell carcinoma, which had originated from the same source as the thymic squamous cell carcinoma (Fig. 2B). In addition, tissues obtained from the lung biopsy were analyzed, but no mutations in the EGFR gene, ALK fusion gene or KIT gene were revealed. At the beginning of September 2013, six cycles of a chemotherapy regimen were initiated, consisting of nab-P and nedaplatin, (nab-P 200 mg on days 1 and 8; nedaplatin 30 mg on days 1–3). Following treatment, the pleural effusion significantly decreased, the lesion in right lung remained stable (Fig. 3C) and the metastatic lesions in the peritoneum were reduced in size (Fig. 3D). The patient demonstrated a good response to nab-P and remains alive three years post-treatment.

## Discussion

Thymic carcinoma is a rare cancer with a poor outcome, as it is usually discovered at an advanced stage. Due to its rarity, the optimal treatment regimen for patients with thymic carcinoma is uncertain. Several aggressive, multimodal treatment strategies have been advocated, but future prospective studies with larger numbers of patients are required in order to validate the most effective therapeutic approach (1–3).

Surgical resection is the preferred curative treatment for thymic tumors, with complete resection conferring the most favorable clinical prognosis (7). At diagnosis, almost 30% of patients present with a locally-advanced tumor, dissemination to the pleura and the pericardium, and/or invasion of adjacent intrathoracic components. Resection of the tumor is usually achieved through a median sternotomy, with removal of the thymus and all adjoining mediastinal fat bordered laterally by the phrenic nerves (8).

Due to the rarity of thymic malignancies, knowledge concerning the outcomes of chemotherapy on these lesions has been based upon retrospective studies, the majority of which are outdated. Furthermore, only a limited number of prospective studies have ever been conducted. The National Comprehensive Cancer Network 2013 guideline on thymomas and thymic carcinomas recommends that first-line treatment should include a cisplatin, doxorubicin and cyclophosphamide (CAP) regimen, a CAP with prednisone, cisplatin, doxorubicin, vincristine and cyclophosphamide regimen, a cisplatin and etoposide regimen, an etoposide, ifosfamide and cisplatin regimen, or a carboplatin and paclitaxel (PC) regimen (9). Second-line chemotherapy is suggested to include etoposide, ifosfamide, pemetrexed, octreotide, prednisone, 5-fluorouracil and leucovorin, gemcitabine or paclitaxel.

In 2011, Lemma *et al* (10) conducted a prospective multicenter study in order to investigate the impact of the PC regimen in patients with advanced and previously untreated thymic carcinoma. The study included 23 patients with thymic carcinoma. Non of the patients were in complete remission, five exhibited partial responses and 12 experienced stable disease. The mean progression-free survival time was five months and the median survival time was 20 months. The PC regimen has moderate clinical activity in patients with thymic malignancies, but this appears to be less than expected when compared with anthracycline-based therapy.

In the last ten years, there have been six studies (11–16) that have reported gene mutations in thymic carcinomas, including those in EGFR exons 18–21, HER-2 exons 19–20, Kras exon 2 and KIT exons 9, 11, 13 and 17. The results of these studies revealed that thymic carcinoma gene mutations are rare. A 2008 study tested for the presence of EGFR and KIT gene mutations by direct sequencing in nine and 11 different thymic cancer tissues, respectively (14). One mutation was identified in exon 11 of the KIT gene, but none in the EGFR gene. Another study, which analyzed 48 cases of thymic carcinoma, identified only six that contained KIT gene mutations: Four within exon 11 (V559A, L576P, Y553 N, W557R), one within exon 9 (E490 K) and one within exon 17 (D820E) (15). Targeted molecular therapy, for example, with the TKI sunitinib, is a new paradigm in the treatment of cancer, which may be useful for patients with thymic carcinoma. However, there have only been three case studies to date describing the success of sorafenib and sunitinib against thymic carcinoma (15,17,18). In the present study, an L858R mutation in exon 21 of the EGFR gene was identified in the tissue of the thymic carcinoma, but the patient did not respond to treatment with TKIs. Furthermore, no mutations were identified in the EGFR, ALK fusion or KIT genes in the metastatic tissues of the lung. Therefore, it was concluded that the EGFR exon 21 L858R mutation in the thymic carcinoma tissue was likely to be a false-positive ARMS result.

Nab-P (also known as Abraxane^®^) has a mean particle size of 130 nm and was originally manufactured with the aim of improving the therapeutic index of paclitaxel (19). In a previous study, nab-P demonstrated a 10-fold increase in the mean concentration of free paclitaxel in the serum compared with solvent-based-paclitaxel (sb-P) alone (19). Nab-P, alone or in combination with carboplatin (nab-P/C), has exhibited positive outcomes in two phase III clinical trials that compared nab-P/C with sb-P plus carboplatin as a first-line treatment for cases of advanced NSCLC (20,21). As a result of these findings, the U.S. Food and Drug Administration approved nab-P/C as a first-line treatment for locally-advanced or metastatic disease in NSCLC patients unsuitable for radiation therapy or curative surgery (19).

To the best of our knowledge, the present study is the first to describe the positive response of a patient with thymic carcinoma to nab-P chemotherapy. As the number of effective treatment options for thymic carcinoma is small, a controlled trial, in which nab-P is analyzed for its clinical efficacy, is required in the future.
